# THE EVALUATION OF INTERPROFESSIONAL EDUCATION IN PRIMARY CARE: A SOCIAL NETWORK ANALYSIS

**DOI:** 10.1093/geroni/igz038.181

**Published:** 2019-11-08

**Authors:** Linda C Smit, Jeroen Dikken, Nienke M Moolenaar, Marieke J Schuurmans, Niek J De Wit, Nienke Bleijenberg

**Affiliations:** 1 University of Applied Sciences Utrecht, Utrech, Netherlands; 2 The Hague University of Applied Sciences, Den Haag, Zuid-Holland, Netherlands; 3 Social and Behavioural Sciences Utrecht University, Utrecht, Utrecht, Netherlands; 4 Education Center, UMC Utrecht Academy, University Medical Center Utrecht, Utrecht, Utrecht, Netherlands; 5 Julius Center for Health Sciences and Primary Care, University Medical Center Utrecht, Utrecht, Utrecht, Netherlands

## Abstract

Effective, safe, person-centred care relies on skilled interprofessional collaboration (IPC) and practice. Little is known about interprofessional education (IPE) to increase IPC in the context of care for frail older people in the community. This study evaluates the effectiveness of IPE on IPC of primary health and social care providers providing care to frail older people in three districts in the Netherlands. A before-after study among 55 health care professionals using social network analysis was performed. The number of contacts increased on average with two contacts. The reciprocity in the districts increased with 15%, 2% and 13%. The diversity of contacts increased between 6% and 10% (p <.001; p .055; p .371). The IPE effectuated a larger, more collaborative, and diverse interprofessional network of health and social care professionals providing care to frail older people suggesting a ripple-effect of networked interventions.

